# Study of Tribological Properties and Evolution of Morphological Characteristics of Transfer Films in PTFE Composites Synergistically Reinforced with Nano-ZrO_2_ and PEEK Particles

**DOI:** 10.3390/polym15173626

**Published:** 2023-09-01

**Authors:** Yuan Qi, Bugong Sun, Yang Zhang, Gui Gao, Peng Zhang, Xiaobao Zheng

**Affiliations:** 1 Mechanical and Electronical Engineering College, Gansu Agricultural University, Lanzhou 730070, China; qiy@gsau.edu.cn (Y.Q.); zhangyang@gsau.edu.cn (Y.Z.); zhangpeng@gsau.edu.cn (P.Z.); zhengxb0915@foxmail.com (X.Z.); 2 State Key Laboratory of Solid Lubrication, Lanzhou Institute of Chemical Physics, Chinese Academy of Sciences, Lanzhou 730000, China; gaogui@licp.cas.cn

**Keywords:** PTFE, PEEK, ZrO_2_ nanoparticles, tribological properties, transfer film, morphological features

## Abstract

The materials tribology community has identified that the transfer film attached to the surface of the counterpart metal during the friction process is not only closely related to the filler modification material but also a key factor affecting the tribological properties of polymer composites; however, there is a lack of feasible methods to quantify the characteristics of the transfer film. In this study, Nano-ZrO_2_ and polyetheretherketone (PEEK) were filled into a PTFE matrix in order to enhance the wear resistance of polytetrafluoroethylene (PTFE). The tribological properties of the modified PTFE composites were tested using a linear reciprocating friction and wear tester, and the entire friction experiment was designed in seven separate stages. Morphological features were extracted and analyzed from photographs of the transfer film acquired by optical microscopy at each friction stage using an image processing program. The thickness and roughness of the transfer film sections were measured using a non-contact profilometer. Abrasive debris were collected, and their morphological features were observed with an electron microscope. The results showed that the synergistic addition of soft PEEK and hard Nano-ZrO_2_ particles effectively inhibited interlayer slippage between PTFE molecular chains, dramatically reducing the size and yield of abrasive debris, and facilitated the improvement of the thickness and firmness of the transfer film, which significantly enhanced the wear resistance of the PTFE composites (the lowest volumetric wear rate for Nano-ZrO_2_/PEEK/PTFE was only 1.76 × 10^−4^ mm^3^/Nm). Quantitative analyses of the morphological characteristics of the transfer films revealed that the coverage and roundness of the transfer films gradually increase with the friction stroke, while the aspect ratio and texture entropy subsequently decrease gradually. The coverage, area, mean, third-order moments, and consistency of the transfer film strongly correlated with the volumetric wear rate (correlation coefficient |r| > 0.9).

## 1. Introduction

PTFE is widely used as a solid lubricating material in space machinery, marine engineering equipment, and medical devices [1,2,3]. This is because PTFE has an excellent low coefficient of friction and properties such as high-temperature resistance, chemical resistance, and biocompatibility. However, PTFE also has drawbacks, the most important of which is its poor wear resistance, severely limiting its use in lubrication applications. Therefore, in recent decades, researchers have been adding modified fillers with different properties (e.g., glass fibers [4,5,6,7], graphite [8,9], metal oxides [10,11], nanoparticles [12,13,14]) to the PTFE matrix to improve the wear resistance of PTFE and have achieved remarkable results. Song et al. [15] investigated the effect of carbon fibers, glass fibers, and MoS_2_ on the tribological properties of PTFE. The results showed that glass fibers as a single filler deteriorate the wear resistance of PTFE composites dramatically. However, the combination of MoS_2_ and glass fibers can synergistically enhance the tribological properties of PTFE. Amenta et al. [16] investigated the ability of glass fiber-reinforced PTFE composites to form thicker and more continuous transfer films on the surface of the counterpart metal when rubbed against uncoated steel but with lower wear resistance than carbon fiber-reinforced PTFE composites. Kandanur et al. [17] found that the volumetric wear rate of PTFE composites with added graphene was 10~30 times lower than that of PTFE composites with the same level of micro-graphite and that the graphene/PTFE debris size was significantly smaller. Burris et al. [18] found that PTFE composites filled with 5% α-Al_2_O_3_ nanoparticles had four orders of magnitude lower volumetric wear rates than unfilled PTFE. Sun et al. [19] systematically investigated the effect of three graphene nanoplates, multilayer graphene, and monolayer graphene on the wear resistance of PTFE composites and found that weakly polymerized strength graphene was 100 times better at reducing wear than strongly polymerized strength graphene. Xiao et al. [20] investigated the effects of various nanofillers (carbon nanopowder, graphene, fullerene, graphite nanopowder, and copper nanopowder) on PTFE composites’ mechanical, thermal, and frictional properties. The experimental results showed that fullerenes can significantly improve the wear resistance of PTFE composites, graphene can increase the stress and strain values of the composites, and all nanofillers can improve the thermal conductivity of PTFE composites.

Polymers as lubricant materials are usually paired with metallic materials to form sliding friction pairs. Due to the high surface energy and hardness of the metal material, the microscopic concave and convex summit on the surface of the counterpart metal produce a scraping effect on the polymer surface during the friction process, resulting in a portion of the material on the polymer body transferring to the counterpart metal surface, aggregating and adhering to it, thus forming a thin film-like medium, also known as a “transfer film” [21,22,23,24,25,26,27]. The transfer film plays a protective and lubricating role in polymeric oil-free friction systems. As a result, the transfer film properties and their interaction with the tribological properties of polymers have been a hot topic of research in tribology in recent years. Ye et al. [28,29] investigated the evolution of the development of transfer films in ultra-low-wear PTFE composites during friction. The results show that factors such as adhesion, chemical properties, abrasive chip morphology, and mechanics of the transfer film all play an essential role in tribological performance. Haidar et al. [30] investigated the correlation between the characteristic size of the transfer film gap (free space length) and the steady state wear rate of the polymer, and the results showed that the free space length is a valid indicator of the quality of the transfer film. Lin et al. [31] concluded that the formation of a running film on the polymer friction surface is indispensable for achieving ultra-low wear and that the transfer film formed on the running film and the counter metal surface plays a synergistic role in enhancing the wear resistance of the polymer. Urueña et al. [32] measured the height and wear of the transfer film using interferometry and profilometry and showed that a thin, uniform, and highly adherent transfer film could form a protective layer with a low shear interface between the PTFE composite and the counter metal surface, thereby reducing the wear rate. Alam et al. [33] concluded that the stability of the transfer film is the key to achieving ultra-low wear rates (10^−7^ mm^3^/Nm) in PTFE composites, that a well-modified filler reduces the size of the abrasive chips contributing to the formation of a stable transfer nucleus, that a stable transfer film reduces the transfer wear rate, and that the stability of the friction interface provides the time required for the friction chemistry to react. The morphological characteristics of transfer films are an essential aspect of interest to researchers and are usually observed, characterized, and analyzed using optical or electron microscopy [34,35,36,37]. Wahl et al. [38] measured and analyzed the thickness of transfer films based on the principle of Newtonian circular interference fringes. Gu et al. [39] found a linear relationship between the thickness of the transfer film and the area of the infrared absorption peak at 1205 cm^−1^, and based on this relationship, the thickness of the transfer film was measured using infrared transmission techniques. Currently, there needs to be a set of quantitative methods to characterize the morphology of the transfer film, as well as a study of the correlation between the quantified transfer film characteristics and the tribological properties of the material.

The aim of this study was to synergistically enhance the tribological properties of PTFE composites by filling them with hard ZrO_2_ nanoparticles and soft PEEK particles. The focus was on the effect of the two soft and hard fillers on the tribological properties and the evolutionary development of the transfer film morphology during the friction process. Based on the theoretical principles of computer graphics, an image processing program for the automatic identification and quantitative analysis of the morphological characteristics of the transfer film was established employing a computer program. The effect of ZrO_2_ nanoparticles and PEEK particles on the morphological characteristics of the transfer film at different friction stages was investigated, as well as determining the correlation between the morphological characteristic parameters of the transfer film and the wear resistance of PTFE composites. These morphological characteristics were used as indicators to evaluate the quality of the transfer film, thus helping to understand the effect of the transfer film on tribological properties.

## 2. Materials and Methods

### 2.1. Specimen Preparation

The three raw materials used in this study were PTFE matrix powder, ZrO_2_ nanopowder, and PEEK particles. PTFE was purchased from Daikin Industries, Ltd. (Osaka, Japan) under the brand name POLYFLON M-18F, with an average particle size of 25 µm and density of 2.16 g/cm^3^. ZrO_2_ nanopowder was purchased from Jiangsu Xianfeng Nanomaterials Technology Co., Ltd. (Nanjing, China) under the brand name XFI01, with an average particle size of 20–40 nm, density of 5.89 g/cm^3^, specific surface area of 12.31 m^2^/g, and purity of 99 wt%. PEEK pellets were purchased from Victrex Ltd. (Lancashire, UK) under the brand name Victrex PEEK 450 PF with an average particle size of 50 µm and density of 1.3 g/cm^3^.

**Figure 1:**
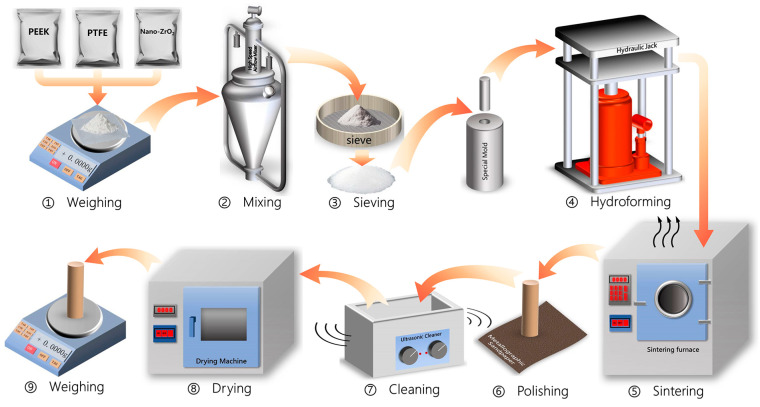
Preparation process of PTFE composite samples.

The preparation process of the PTFE composites is shown in Figure 1. The ZrO_2_ nanoparticles, PEEK particles, and PTFE powder were weighed and formulated using an electronic balance (with an accuracy of 0.1 mg) according to the formulation proportions shown in Table 1. The formulated raw materials were first mixed mechanically using a high-speed airflow mixer. Then, the mixture was sieved using a 150-mesh sieve to remove agglomerated particles from the mixture so that the filler modifier and PTFE powder could be mixed adequately and uniformly. The mixed raw material powder was filled into special molds (cylindrical cavities with a diameter of 6 mm), which were placed in a hydraulic press, and the material was cold pressed at a pressure of 45 MPa for the forming operation. The pressed cylindrical samples were sintered in the sintering furnace according to a pre-set temperature control procedure (sintering temperature set at 375 °C, heating rate of 2 °C/min, kept warm for 120 min, then cooled naturally to room temperature). The end surfaces of the sintered cylindrical samples were sanded with 2000-grit metallographic paper to ensure good contact with the surface of the counterpart metal. The polished samples were soaked in acetone solvent for ultrasonic cleaning, then dried in a drying oven before being weighed on an electronic balance and recorded in a register.

### 2.2. Reciprocal Friction and Wear Test

**Figure 2:**
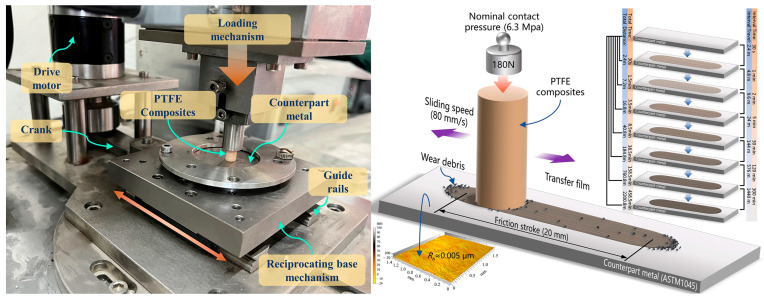
Structure of the reciprocating friction and wear tester.

Each PTFE composite was tested in seven separate stages (30 s, 1 min, 2 min, 5 min, 30 min, 2 h, and 5 h for each stage) on a linear reciprocating friction and wear tester (Model LSM-200, ZKKH Technology Development Co., Lanzhou, China), with a cumulative friction test duration of 458.5 min (cumulative friction stroke of 2200.8 m), to observe the evolution of the tribological properties and transfer film characteristics at each stage of friction, as shown in Figure 2.

The normal load of 180 N was applied to the top of the specimen (nominal contact pressure of approximately 6.3 MPa), the friction stroke was 20 mm, the motor speed was 120 r/min, and the ambient temperature was 25 °C. After each stage of the friction test, the sample and the counterpart metal were carefully removed from the friction testing machine, and the following observations were made:

The specimens were weighed using an electronic balance, then the mass loss and the average volumetric wear rate *K* of the specimens in each section of the test were calculated.

(1) $$K=\frac{\Delta m}{\rho NL}$$

where ∆*m* is the wear weight, *ρ* is the specimen’s actual density, *N* is the normal load applied to the sample, and *L* is the total sliding distance.

High-resolution images (magnification from 50× to 1000×, image resolution 2560× 1920 pixels) of the surface of the paired metal were obtained using an optical microscope (Axio Imager M2m, ZEISS, Jena, Germany) to provide raw image information for subsequent morphological characterization of the transfer film. Figure 2 shows a high-resolution image of the complete transfer film stitched together from partial images of the transfer film at 50× magnification.

A non-contact 3D profiler (MicroXAM-800, KLA-Tencor, Milpitas, CA, USA) was used to obtain the thickness and roughness of the transfer film on the counterpart metal surface at each friction stage.

After completing the above observations, the specimen and counterpart metal were reinstalled on the friction tester, and the next stage of the friction wear test was carried out. It was important to note that during the dismantling, testing, and mounting process, it was strictly necessary to ensure that the friction surfaces of the specimen and the counter metal were not in direct contact with other substances to avoid contamination and damage to the formed transfer film. At the end of the test, the collected debris was soaked in a dispersant for ultrasonic dispersion. The debris was sprayed with gold before observation by scanning electron microscopy (Quanta FEG450, FEI, Eindhoven, The Netherlands).

### 2.3. Transfer Film Image Feature Recognition and Extraction Method

#### 2.3.1. Preprocessing of Transfer Film Images

**Figure 3:**
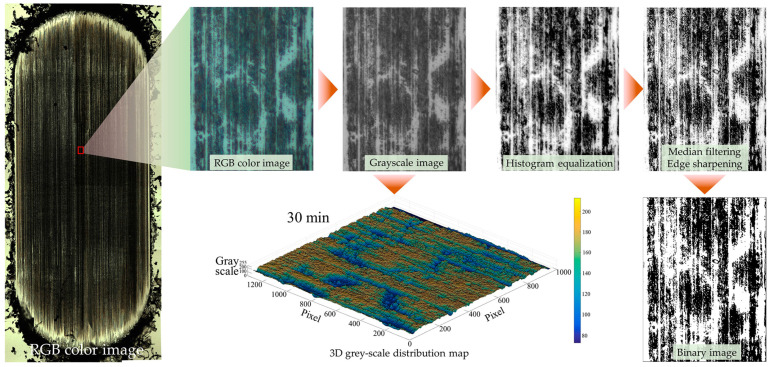
Pre-processing flow of the transfer film image [40].

The images of the transfer film acquired by optical microscopy were RGB images (each pixel point can present 256^3^ colors). The transfer film RGB image was first converted into a grey-scale image (each pixel point contains only grey-scale information ranging from 0~255). Then, a 3D distribution of the grey-scale values of the transfer film was generated with the help of a visualization program. These images needed to be pre-processed [40]. The pre-processing flow of the transfer film image is shown in Figure 3. The purpose of pre-processing was to reduce noise, suppress aberrations and distortions in the raw image, and enhance the contrast between the areas covered by the transfer film and the bare areas of the counterpart metals not covered by the transfer film, thus facilitating the segmentation and extraction of the transfer film image by subsequent image processing programs. Image pre-processing included image space transformation, image enhancement, image edge smoothing, image filtering, and sharpening.

Image pre-processing techniques of the transfer film used in this paper consisted of the following steps.

Image-space transformation: The raw color image of the transfer film was converted into a greyscale image.

Histogram equalization: The individual grey levels in the image had the same probability, and therefore the entropy in the image had a maximum value.

Median filtering: Used to enhance the contrast of the area covered by the transfer film under certain conditions, effectively suppress the noise in the background area not covered by the transfer film, and overcome the problem of blurred details in images processed by linear filtering methods.

Image edge sharpening: The transfer film edges were sharpened using the Laplace operator to enhance the detailed features and contrast of the transfer film edges.

#### 2.3.2. Transfer Film Image Segmentation and Edge Detection

Based on the grey-scale characteristics of the image, the grey-scale image of the transfer film was segmented into a target area (the area covered by the transfer film) and a background area (the bare surface of the dyadic metal) using the Otsu thresholding method [41]. The grey-scale values of the target region are all less than or equal to the preset segmentation threshold T. In comparison, the grey-scale values of the background region are all greater than this threshold T. This paper automatically calculates the optimal segmentation threshold in the image using the maximum inter-class difference method. This paper used the maximum inter-class difference method to calculate the best segmentation threshold in the image automatically.

Determining the target region boundary in the transfer film image is the key to image feature extraction. According to the principles of computer graphics and mathematical set theory, a boundary in an image is a collection of connected pixels on the boundary of two regions with distinctly different characteristics, reflecting jumping changes in the grey-scale of the boundary. Discontinuous features of the grey value on the boundary (i.e., whether a pixel point in the image is a boundary point of the target region) can be detected by calculating the derivative. In this paper, the calculus Sobel operator method was used to detect the edge contours of the transfer film. The Sobel operator is able to smooth out the noise and provide more accurate directional information about the edges of the target region in the image so that better results can be obtained for images with gradual grey- scale changes and much noise [42].

#### 2.3.3. Morphological Processing of Binary Images of Transfer Films

The basic principle of morphological image processing is to use a structural element (also known as a pixel probe) to move continuously through the image to check whether the structural element is entirely inside the target area, thereby determining the interrelationship between regions in the image and understanding the structural features of the image. Morphological image processing consists of the following four basic operations:

(1) Expansion: A morphological operation that blends elements of the background area near the boundary of the target area into the target area.
(2) Erosion: A morphological operation that removes the boundary points of the target area from the image.
(3) Open operation: Erosion operation followed by expansion operation.
(4) Closed operation: The expansion operation is performed first, then the erosion operation is performed.

These four basic operations of morphological image processing can remove the convex corners of the target area boundaries, fill in the concave corners and voids in the target area, suppress the noise in the background area, and improve the smoothness of the boundaries, thus facilitating the acquisition of the key shape features in the target area.

#### 2.3.4. Quantification of Image Features of Transfer Films

The morphological features quantified from the transfer film images in this paper included shape features and texture features.

The shape feature is a global or local feature used to describe the shape of the transfer film in computer graphics theory. Shape features can be divided into two categories: one is the contour feature that describes the shape of the object’s boundary, and the other is the regional feature that describes the internal shape of the object. Table 2 lists five shape feature parameters, definitions, and calculation formulas.

Texture features reflect structures with slow or periodic changes in the image. Texture features are represented by sequential repetition of the local structure of the image, random arrangement of structural features, and roughly uniform consistency. Based on the statistical data provided by the grey-scale histogram, the texture information of an image, including interval distance, direction, magnitude of change, and speed of change, was quantified and described by calculating the correlation of the grey-scale values between two points in the image at a certain distance and direction. Table 3 lists this paper’s four texture feature parameters, definitions, and calculation formulas.

## 3. Results and Discussion

### 3.1. Synergistic Enhancement of Tribological Properties of PTFE Composites by Nano-ZrO_2_ and PEEK

**Figure 4:**
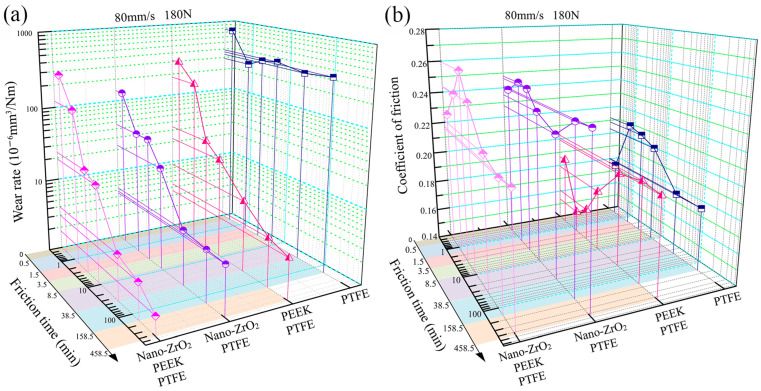
Tribological behaviours of the PTFE composites filled with different volume contents of PEEK and Nano-ZrO_2_ particles: (**a**) Wear rate, (**b**) friction coefficient.

**Figure 5:**
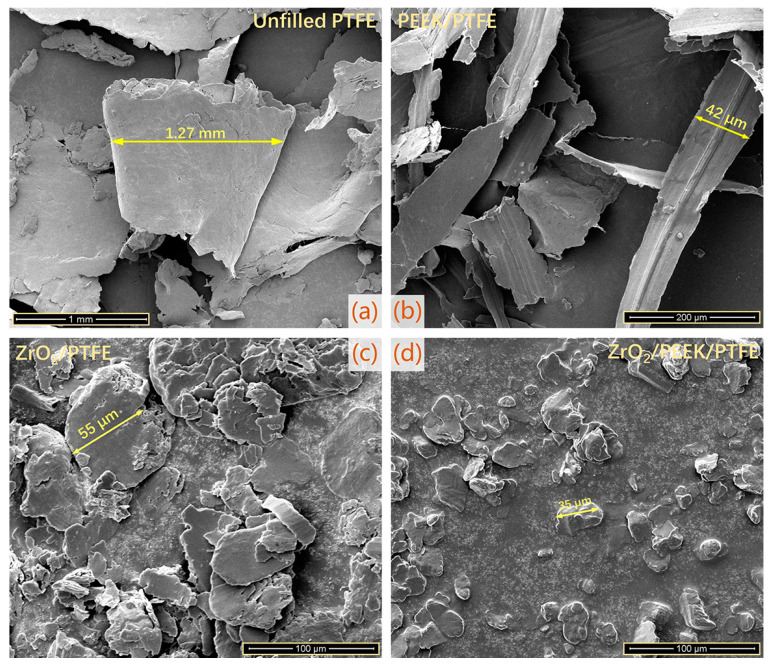
The morphological characteristics of debris produced by PTFE composites: (**a**) Unfilled PTFE, (**b**) PEEK/PTFE, (**c**) ZrO_2_/PTFE, (**d**) ZrO_2_/PEEK/PTFE.

**Figure 6:**
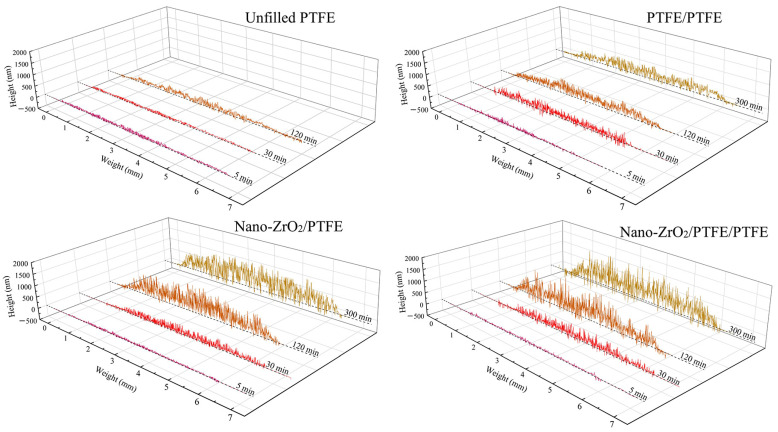
Evolution of the cross-sectional thickness of the transfer film.

The pure PTFE exhibited a very high wear rate during the friction process, especially in the first experimental phase (0~30 s), where the volumetric wear rate was as high as 7.7 × 10^−4^ mm^3^/Nm. In the subsequent experimental phases, the volume wear rate of pure PTFE remained at a high level of 3 × 10^−4^ mm^3^/Nm. The extremely inferior wear resistance of pure PTFE resulted in the frictional wear test being carried out for only the first six stages, with a cumulative friction time of 158.5 min (cumulative frictional travel of 760.8 m), as shown in Figure 4a. On the other hand, pure PTFE exhibited excellent lubricity during the 5th and 6th friction stages (720 m of friction travel for both stages combined) with an average coefficient of friction of only 0.18, as shown in Figure 4b. Pure PTEF continuously threw large amounts of flaky debris of millimeter size out of the friction system during the friction process, as shown in Figure 5a. The thickness of the transfer film adhering to the surface of the counterpart metal rubbed against pure PTFE was very low and remained almost unchanged throughout the friction process, as shown in Figure 6. The key reason for the excellent lubricity and significantly inferior wear resistance of pure PTFE is the carbon–carbon molecular chain structure of PTFE. This molecular chain structure has a carbon backbone with fluorine atoms wrapped around the outside. The repulsive forces between the fluorine atoms on the different molecular chains make the chains susceptible to interlayer slippage movement, which reduces the shear strength of the PTFE matrix and generates a large number of crisp debris.

The volumetric wear rates of all three PTFE composites filled with Nano-ZrO_2_ and PEEK showed a gradual decrease throughout the friction process, as shown in Figure 4a. The volumetric wear rate of PEEK/PTFE gradually decreased from over 1 × 10^−4^ mm^3^/Nm at the initial friction stage to 1 × 10^−5^ mm^3^/Nm at the stable friction stage. The lowest value of PEEK/PTFE volumetric wear rate was 3.53 × 10^−6^ mm^3^/Nm. The average coefficient of friction of PEEK/PTFE was lower than that of pure PTFE in the early stages and higher than that of pure PTFE in the stable friction stage. Unlike the flakey debris of pure PTFE, PEEK/PTFE produced predominantly ribbon-like debris and had a much smaller width dimension than that of PTFE, as shown in Figure 5b.

The volume wear rate of Nano-ZrO_2_/PTFE decreased significantly during the first four experimental stages, while it leveled off and remained below 1 × 10^−5^ mm^3^/Nm during the last three experimental stages. The lowest value of volumetric wear rate for Nano-ZrO_2_/PTFE was 4.56 × 10^−6^ mm^3^/Nm. The average coefficient of friction of Nano-ZrO_2_/PTFE varied relatively little over the seven friction stages, remaining at around 0.24. In the stable friction stages, the volume wear rate of Nano-ZrO_2_/PEEK/PTFE was only 1.76 × 10^−6^ mm^3^/Nm, showing the best wear resistance of the four specimens. The average coefficient of friction of Nano-ZrO_2_/PEEK/PTFE at the later stages of the experiment was 0.22, lower than the 0.24 of Nano-ZrO_2_/PTFE for the same period. Both Nano-ZrO_2_/PTFE and Nano-ZrO_2_/PEEK/PTFE friction produced debris with a powdery morphological character, and the size of Nano-ZrO_2_/PEEK/PTFE debris was smaller, as shown in Figure 5c,d. During the stable friction phase, the average thickness of the transfer film formed by Nano-ZrO_2_/PTFE and Nano-ZrO_2_/PEEK/PTFE on the surface of the counterpart metal exceeded 500 nm, significantly higher than the transfer film thickness of pure PTFE and PEEK/PTFE, as shown in Figure 6. This indicates that the hard Nano-ZrO_2_ particles dispersed in the PTFE matrix acted as a backbone to support and enhance the creep resistance, compressive strength, and hardness of the PTFE composites, thus effectively inhibiting the interlayer slip between the carbon–carbon molecular chains of PTFE. The soft PEEK particles in Nano-ZrO_2_/PEEK/PTFE act as a link between the hard Nano-ZrO_2_ particles and the PTFE matrix, thus exhibiting both optimum wear resistance and better lubricity than Nano-ZrO_2_/PTFE.

### 3.2. The Morphological Evolution of the Transfer Film

**Figure 7:**
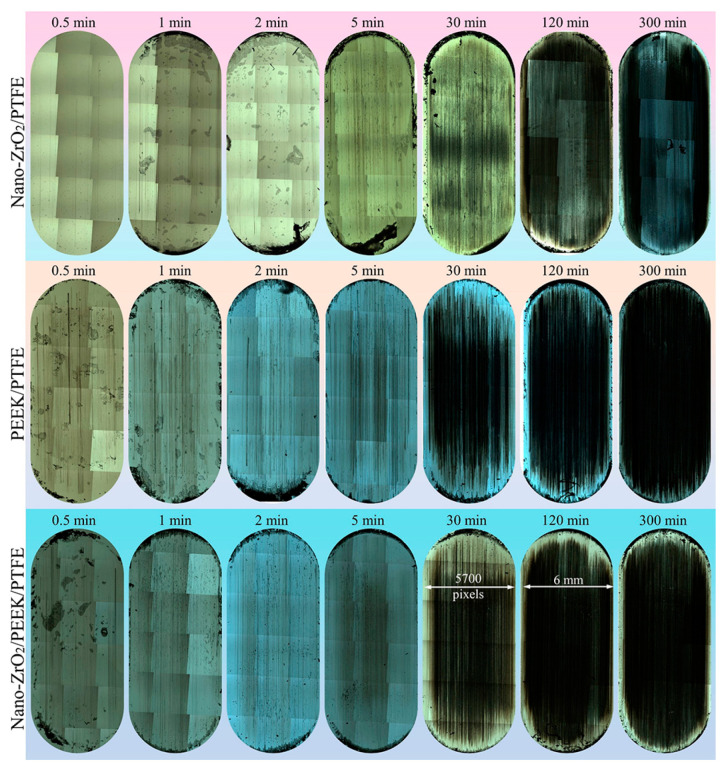
True color optical images of transfer films at various friction stages.

Figure 7 shows optical images of the complete transfer films of the three PTFE composites over the entire friction area.

At 30 min, the transfer films formed by Nano-ZrO_2_/PTFE and Nano-ZrO_2_/PEEK/PTFE covered most of the friction area and showed noticeable scuff marks. In contrast, the transfer films formed by PEEK/PTFE during the same period had a smaller area, indicating that the nanoparticles were beneficial in increasing the formation rate of the transfer film. In the later stages of the friction experiments, the transfer films formed by all three materials covered the entire friction area, showing good coverage.

**Figure 8:**
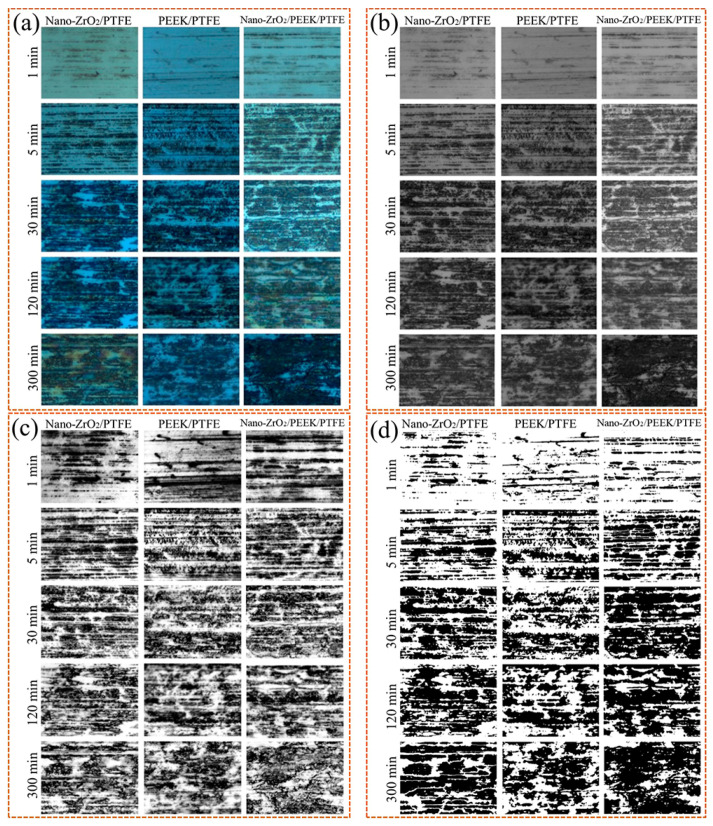
Pre-processing of transfer film images: (**a**) Original color image, (**b**) grey-scale images, (**c**) median filtered image, (**d**) binary image.

**Figure 9:**
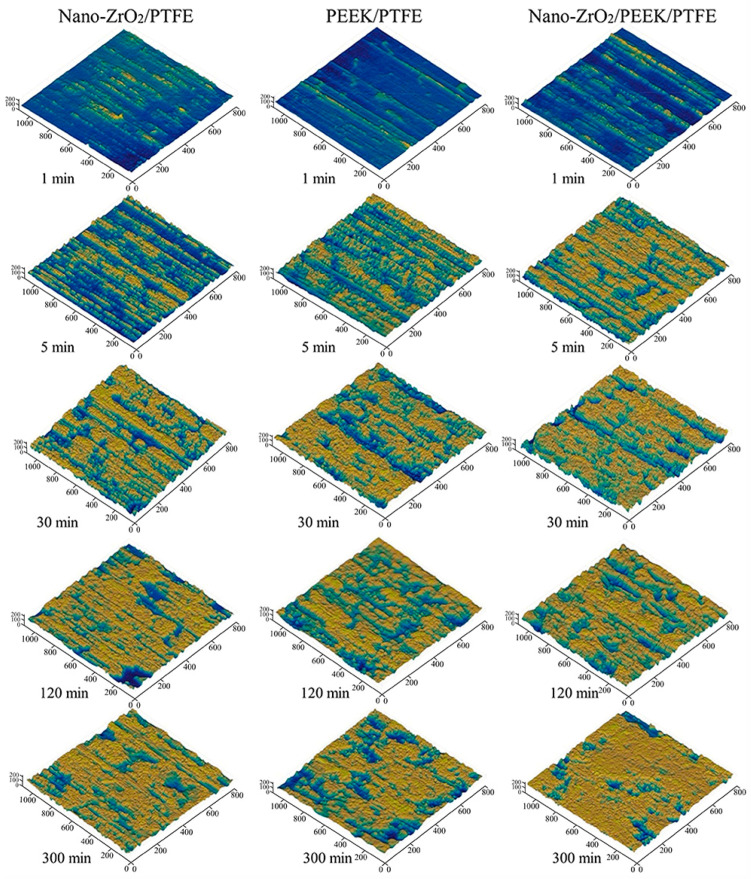
Three-dimensional grey-scale morphology of the transfer film.

Figure 8 shows the morphological characteristics and evolution of the local details of the transfer film. Figure 9 shows the 3D grey-scale image of the transfer film, which can be generated by computer processing of the transfer film image obtained from the optical microscope, and is, therefore, simpler and more efficient than using a 3D profiler to obtain the surface shape of the transfer film. Comparing the three sets of samples at 1 min in Figure 8 and Figure 9, it can be seen that at the beginning of the friction period, some transfer film had formed on the surface of the counterpart metal rubbed against PTFE composites filled with Nano-ZrO_2_. In contrast, the surface of the PEEK/PTFE counterpart was relatively clean, with minimal transfer film covering it during the same period. At the same time, Nano-ZrO_2_/PEEK/PTFE had the best transfer film coverage among the three. By the end of the rubbing period, the transfer film thickness and distribution uniformity of Nano-ZrO_2_/PEEK/PTFE were significantly better than those of the other two materials.

### 3.3. Quantitative Analysis of the Evolution of the Morphological and Textural Characteristics of Transfer Films

**Figure 10:**
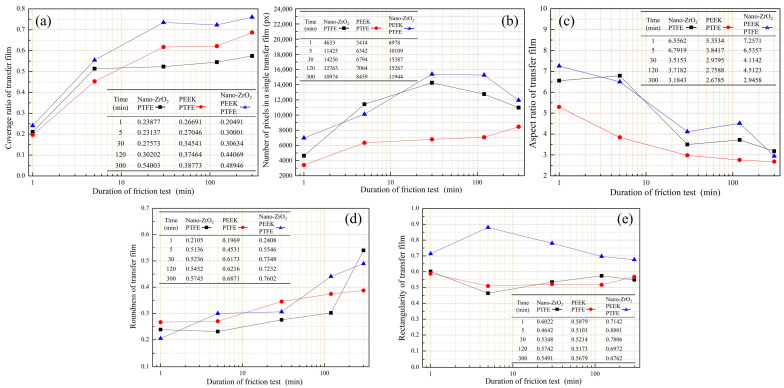
Geometry characteristics of transfer film. (**a**) Coverage ratio; (**b**) area; (**c**) aspect ratio; (**d**) roundness; (**e**) rectangularity.

The coverage area of the transfer film and coverage ratio are quantitative indicators to measure the integrity of the transfer film on the surface of the counterpart metal. All three groups of PTFE-reinforced materials showed a gradual increase in transfer film area coverage with the growth of the friction stroke, and the growth rate was faster in the early stages of friction and gradually stabilized in the later stages, as shown in Figure 10a. The transfer film area coverage of Nano-ZrO_2_/PEEK/PTFE was 0.55 when the friction test was carried out for up to 5 min. The transfer film coverage of Nano-ZrO_2_/PTFE and PEEK/PTFE during the same period was 0.51 and 0.45, respectively, indicating that the hard nanoparticles contribute to the rapid formation of a transfer film on the surface of the counterpart metal at the beginning of the friction. The transfer film coverage of Nano-ZrO_2_/PTFE was lower than that of the other two PTFE composites filled with PEEK particles at the same period when the friction test lasted up to 30 min, due to the relatively soft PEEK particles facilitating enhanced transfer film firmness, thus maintaining the integrity of the composite on the surface of the counterpart metal. Nano-ZrO_2_/PEEK/PTFE exhibited the best transfer film coverage at different stages of the friction experiments, and it also had the lowest wear rate of the three PTFE composites over the same period, suggesting that the hard nanoparticles and the soft PEEK filler can synergistically improve the transfer film coverage as well as the wear resistance.

Comparing two counterpart metal surfaces with the same value for the coverage of transfer film, if the average area of individual transfer films covering one of the surfaces is smaller, the greater the number of individual transfer films. Figure 10b shows the trend in the average area of the individual transfer film for the three PTFE composites at different friction stages. Although the area of the individual transfer film of PEEK/PTFE maintained a continuous and slow growth trend throughout the friction process, its area was significantly smaller than that of the transfer film with nanoparticles added in the same period. This phenomenon indicated that the transfer film distribution of PEEK/PTFE was dispersed relatively and that the PEEK particles were not conducive to linking separate transfer films. The individual transfer film areas of Nano-ZrO_2_/PTFE and Nano-ZrO_2_/PEEK/PTFE showed a trend of increasing first and then decreasing. This trend in the transfer film also corroborated the change in wear rate, indicating that the hard nanoparticles scrape the already-formed transfer film in the later stages of friction and split the larger transfer film into smaller ones.

The aspect ratios of the transfer films of all three PTFE composites gradually decreased as the frictional stroke increased, indicating that the transfer films were gradually fusing and expanding, as shown in Figure 10c. On the other hand, the aspect ratios of both nanoparticle-containing transfer films were higher than those of the PEEK/PTFE transfer films during the same period, probably due to the scraping action of the hard nanoparticles cutting the transfer film along the friction direction.

The degree of circularity can measure the similarity of the transfer film boundary profile to a circle. The greater the roundness value, the greater the similarity of the transfer film contour to a circle. As shown in Figure 10d, the roundness values for all three transfer films gradually increased with increasing friction stroke, indicating that the edges of the transfer films gradually became smoother. The effect of the two filler materials on roundness also differed. In the early stages of the friction test, the PEEK particles were more beneficial in improving the roundness of the transfer films. In contrast, in the later stages of the test, the nanoparticles showed a more significant effect on improving the roundness values.

The similarity between the boundary profile of the transfer film and its outer rectangle can be measured by the degree of rectangularity. The rectangularity of the Nano-ZrO_2_/PEEK/PTFE transfer film was higher than that of the other two transfer films at all friction stages. However, the rectangularity of the three materials did not show a significant trend throughout the friction process, as shown in Figure 10e.

**Figure 11:**
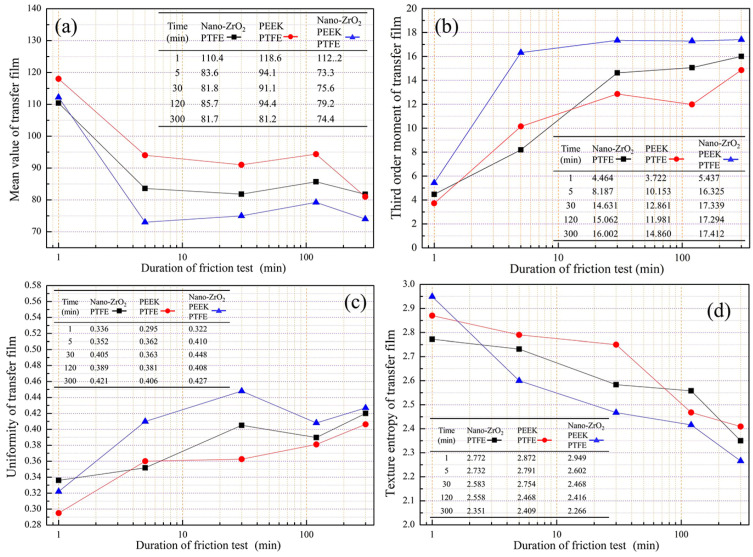
Geometry characteristics of transfer film. (**a**) Coverage ratio; (**b**) area; (**c**) aspect ratio; (**d**) roundness.

The mean value is a quantitative measure of the average luminance of the pixels in a transfer film image. As the reflectivity of the metal material is stronger than that of the transfer film, a lower mean value indicates a smaller area of exposed counterpart metal. At the initial stage of friction, the mean values of all three transfer films showed a significant decreasing trend, then the changing trend became stable, as shown in Figure 11a. At 1 min, the average value for PEEK/PTFE was higher than the other two materials, indicating that the transfer film of PEEK/PTFE was produced at a slower rate during this period, thus leaving a larger area of bare metal on the surface of the counterpart metal. At 300 min, the mean value of PEEK/PTFE was very close to that of Nano-ZrO_2_/PTFE, and both were higher than that of Nano-ZrO_2_/PEEK/PTFE. For the rest of the friction period, the mean value of Nano-ZrO_2_/PEEK/PTFE was the lowest among the three transfer films, followed by Nano-ZrO_2_/PTFE, and the mean value of PEEK/PTFE was higher than the former two.

The third-order moment measures the degree of skewness of the color components (color asymmetry) in the transfer film image. As shown in Figure 11b, the third-order moments for all three transfer films were greater than 0. They showed a gradually increasing trend, indicating that the color component of the image was gradually skewed to the right. In the same friction phase, the nanoparticles were more beneficial in increasing the third-order moments compared to PEEK.

Uniformity is a quantitative measure of the smoothness of the transfer film texture, with higher values indicating a smoother texture and lower values indicating a rougher texture. The uniformity of the PEEK/PTFE transfer film showed a trend of continuous growth with the increase of the friction stroke, indicating that the texture of the transfer film was rough at the beginning of the friction, then gradually evolved towards a smooth texture, as shown in Figure 11c. The uniformity of PEEK/PTFE was lower than that of the other two materials filled with nanoparticles at the same friction stage, so the texture appears rougher. The uniformity of the two transfer films with added nanoparticles showed an increasing trend at the beginning of the friction. However, the value of uniformity remained stable at around 0.4 after 30 min because the transfer film was continuously scraped by the nanoparticles that have accumulated on the friction interface, thus inhibiting further growth in texture smoothness.

The texture entropy reflects the change in greyscale in the transfer film image, with higher texture entropy indicating a greater complexity of grey-scale changes in the image and vice versa. The texture entropy of all three material transfer films showed a gradual decrease with increasing friction stroke, which indicates that the complexity of the grey-scale of the transfer film was also gradually decreasing, as shown in Figure 11d.

The analysis focused on examining the correlation between the morphological characteristics of the transfer film formed at various friction stages and the volumetric wear rate, using the calculation formula of Pearson’s correlation principle expressed as follows:

(2) $$r=\frac{\sum_{i-1}^{n}\left(x_{i}-\bar{x}\right)\left(y_{i}-\bar{y}\right)}{\sqrt{\sum_{i-1}^{n}{\left(x_{i}-\bar{x}\right)}^{2}\sum_{i-1}^{n}{\left(y_{i}-\bar{y}\right)}^{2}}}$$

where “*x*” represents the morphological characteristics of the transfer film, “*y*” represents the tribological properties of the PTFE composites at different friction stages, and “*r*” denotes the correlation coefficient between “*x*” and “*y*”. 

In correlation analysis, a correlation coefficient (|*r*|) greater than 0.95 is considered significant, while a value equal to or greater than 0.8 indicates a high correlation. On the other hand, a correlation coefficient falling within the range of 0.5 to less than 0.8 is classified as moderate, while values between 0.3 and 0.5 indicate a low correlation. Lastly, a correlation coefficient below 0.3 signifies no correlation between the variables under examination.

The results of the studies in Table 4 show a certain regularity in the evolution of the morphological characteristics of the transfer film of PTFE composites during friction and that hard nanoparticles and soft PEEK particles play a different role in influencing this change in the transfer film. There was a clear relationship between the volumetric wear rate and some morphological characteristics of the transfer film at different friction stages. The coverage of the transfer film, as well as its individual area, mean value, third−order moments, consistency, and textural entropy, showed a strong correlation with the wear rate. Overall higher coverage, smoother edge profiles, and consistent textural characteristics contributed to the wear resistance of PTFE composites. Therefore, these five morphological characteristics could be chosen to evaluate the film-forming quality of the transfer film quantitatively and be used as an indirect basis for evaluating the wear resistance of PTFE composites.

## 4. Conclusions

In this work, the mechanism of the enhancing effect of PEEK and nanoparticles on the wear resistance of PTFE composites was investigated, as well as the evolution of tribological properties and morphological characteristics of the transfer film during the friction process. The following conclusions were drawn.

(1) Pure PTFE generated many flakes of considerable size (millimeter scale) during friction due to the slip motion between the carbon–carbon molecular chains, resulting in low wear resistance (3 × 10^−4^ mm^3^/Nm). Adding PEEK particles and ZrO_2_ nanoparticles to the PTFE matrix effectively suppressed interlayer slip and improved the shear deformation resistance of the matrix. The hard ZrO_2_ nanoparticles were highly efficient at cutting through debris detached from the matrix, resulting in a noticeable reduction in the size (micron scale) and the number of abrasive chips and, therefore, a significant improvement in the wear resistance of the PTFE composite (10^−6^ mm^3^/Nm). In particular, the volumetric wear rate of Nano-ZrO_2_/PEEK/PTFE was only 1.76 × 10^−6^ mm^3^/Nm at the late friction stage.
(2) The PEEK and nanoparticles facilitated the formation of a firm and stable transfer film on the surface of the counterpart metal, and the nanoparticles, in particular, greatly increased the thickness of the transfer film during the stable friction phase, which also resulted in a higher coefficient of friction compared to pure PTFE. The soft PEEK particles dispersed in the matrix could effectively encapsulate the hard nanoparticles, which acted as a link between the PTFE matrix and the nanoparticles, so the increase in friction coefficient was not as large as that of Nano-ZrO_2_/PTFE.
(3) The morphological characteristics of the transfer film evolved dynamically during the friction process, in which the transfer film’s coverage, roundness, third-order moment, and consistency showed an increasing trend. In contrast, the aspect ratio and texture entropy demonstrated a gradual decrease. The trend of these morphological characteristics indicated that the transfer film gradually fused during the friction process, and the haphazardness of the transfer film shape gradually decreased. The correlation analysis showed that the five morphological characteristics of transfer film coverage, area, mean value, third-order moment, and consistency exhibited a significant correlation with the volume wear rate of the PTFE composite.

## Figures and Tables

**Table 1:** Formulation ratio of PTFE composites.

Material	PTFE (Vol.%)	PEEK (Vol.%)	Nano-ZrO_2_ (Vol.%)
Pure PTFE	100	0	0
PEEK/PTFE	90	10	0
Nano-ZrO_2_/PTFE	92	0	8
Nano-ZrO_2_/PEEK/PTFE	82	10	8

**Table 2:** Definitions and calculation formulas of shape feature parameters of the transfer film.

Shape Feature	Definition	Calculation Formula
Perimeter of the transfer film (*L*) 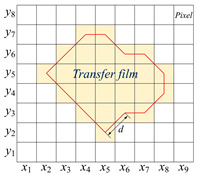	The transfer film boundary’s pixel points are arranged to form a closed boundary. The Euclidean distance *d* between two adjacent pixel points on the boundary is calculated in turn using the 8-chain code method, and the sum of the distances *d* is the perimeter of the transfer film boundary *L*.	$\begin{array}{l} d=\sqrt{{\left(x_{1}-x_{2}\right)}^{2}+{\left(y_{1}-y_{2}\right)}^{2}}\\ L=\sum d \end{array}$
Area covered by transfer film (*A*_1_) 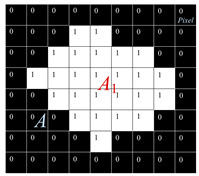	The target area is displayed in white with a pixel value of 1. The background area is black with a pixel value of 0. The sum of the number of pixels in the image with a pixel value of 1 is calculated as the area covered by the transfer film.	$A_{1}=\overset{m}{\underset{i=1}{\sum }}\overset{n}{\underset{j=1}{\sum }}I(x_{i},y_{i})$
Coverage ratio of transfer film (*r*)	The ratio of the area covered by the transfer film to the area not covered by the transfer film.	$r=\frac{A_{1}}{A}$
Roundness of transfer film (*C*)	*C* reflects the degree to which the target area is close to a circle. A higher value of roundness indicates that the shape of the transfer film area is closer to a circle.	$C=\frac{4\pi A_{1}}{L^{2}}$
Rectangularity of transfer film (*R*) 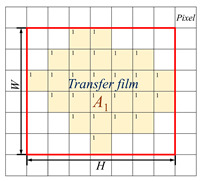	The ratio between the area of the target region and the area of its smallest external rectangle. *R* reflects how close the shape of the transfer film is to a rectangle.	$R=\frac{A_{1}}{W\times H}$
Remarks	*x* and *y* are the values of the horizontal and vertical coordinates of the pixel points respectively. *I* is the pixel value *A* is the total number of pixel points in the image. *W* and *H* represent the width and height of the transfer film area respectively. $W=\max\left(\sum_{j=1}^{n}I\left(x_{j},y_{j}\right)\right)$ $H=\max\left(\sum_{i=1}^{m}I\left(x_{i},y_{i}\right)\right)$	

**Table 3:** Definitions and calculation formulas of texture feature parameters of the transfer film.

Textural Feature	Definition	Calculation Formula
Mean value of transfer film (*M*)	*M* is the average luminance value of all pixels in the area of the transfer film, which reflects the overall brightness of the transfer film.	$M=\overset{G-1}{\underset{i=0}{\sum }}z_{i}p(z_{i})$
The third-order moment of transfer film (*μ*_3_)	*μ*_3_ measures the skewness of the grey-scale histogram of the transfer film image and thus determines the symmetry of the histogram.	$\mu_{3}=\sum_{i=0}^{G-1}{\left(z_{i}-M\right)}^{3}p\left(z_{i}\right)$
Uniformity of transfer film (*U*)	*U* reflects the smoothness of the texture in the transfer film image. A higher value of uniformity indicates a smoother texture in the image; conversely, a lower uniformity indicates a rougher image.	$U=\overset{G-1}{\underset{i=0}{\sum }}p^{2}(z_{i})$
Texture entropy of transfer film (*E*)	*E* reflects the variability and complexity of the texture in a transfer film image. A higher entropy value indicates a more complex transfer film image; conversely, a lower entropy value indicates a less complex image.	$E=-\overset{G-1}{\underset{i=0}{\sum }}p(z_{i})\log_{2}p(z_{i})$
Remarks	*z*_i_ is a random variable in the grey level of the image. *G* is the grey level of the image. *P*(*z*_i_) is the corresponding grey histogram of the image (*i* = 0, 1, …, *G* − 1). *μ*_2_ is the second-order moment of the mean value *M*.	

**Table 4:** Correlation of transfer film morphological characteristics parameters with volumetric wear rate.

Morphological Characteristics of the Transfer Film		Nano-ZrO_2_ PTFE	PEEK PTFE	Nano-ZrO_2_ PEEK PTFE
Geometrical characteristics	Overall coverage ratio	0.991742451	0.995184464	0.970536919
	Area of the individual transfer film	0.903301132	0.931414003	0.932489267
	Aspect ratio	0.7468347327	0.8370890552	0.788524198
	Roundness	0.529314952	0.682693702	0.755553724
	Rectangularity	0.235274832	0.713107776	0.113152496
Textural features	Mean value	0.901197369	0.933141344	0.969658099
	Third−order moments	0.976515695	0.945376412	0.998476153
	Consistency	0.912254056	0.921862415	0.952934061
	Texture entropy	0.792752312	0.642134468	0.932279773

## Data Availability

Not applicable.

## References

[1] Speerschneider C.J., Li C.H. (1962). Some observations on the structure of polytetrafluoroethylene. J. Appl. Phys..

[2] Bunn C.W., Cobbold A.J., Palmer R.P. (1958). The fine structure of polytetrafluoroethylene. J. Polym. Sci..

[3] Meng Y., Xu J., Jin Z., Prakash B., Hu Y. (2020). A review of recent advances in tribology. Friction.

[4] Gweon J.H., Joo B.S., Jang H. (2016). The effect of short glass fiber dispersion on the friction and vibration of brake friction materials. Wear.

[5] Huang R., Ma S., Zhang M., Yang J., Wang D., Zhang L., Xu J. (2019). Wear evolution of the glass fiber-reinforced PTFE under dry sliding and elevated temperature. Materials.

[6] Bandaru A.K., Kadiyala A.K., Weaver P.M., O’ Higgins R.M. (2020). Mechanical and abrasive wear response of PTFE coated glass fabric composites. Wear.

[7] Birleanu C., Marius P., Mircea C., Bere P., Glad C., Cristian D., Filip D. (2023). Tribo-mechanical investigation of glass fiber reinforced polymer composites under dry conditions. Polymers.

[8] Li Y., Chen Y., Guo Y., Bian D., Zhao Y. (2022). Tribological behavior of PEEK/PTFE composites reinforced with carbon fibers and graphite. Materials.

[9] Bhargava S., Makowiec M.E., Blanchet T.A. (2020). Wear reduction mechanisms within highly wear-resistant graphene- and other carbon-filled PTFE nanocomposites. Wear.

[10] Vasilev A., Nadezhda L., Struchkova T.S., Okhlopkova A.A., Danilova S.N. (2023). Mechanical and tribological properties of polytetrafluoroethylene modified with combined fillers: Carbon fibers, zirconium dioxide, silicon dioxide and boron nitride. Polymers.

[11] Lei F., Hu K.A., Li J.L., Zhao B.Y. (2001). The friction and wear characteristics of nanometer ZnO filled polytetrafluoroethylene. Wear.

[12] Yuan Q., Gong J., Cao W.H., Wang H.G., Ren J.F., Gao G. (2018). Tribological behaviour of PTFE composites filled with PEEK and nano-Al_2_O_3_. Tribol. Trans..

[13] Wang Q.H., Xue Q.J., Shen W.C. (1997). The friction and wear properties of nanometre SiO_2_ filled polytetrafluoroethylene. Tribol. Int..

[14] Alam K.I., Bragaw P., Burris D.L. (2022). Isolating the tribochemical and mechanical effects of nanofillers on PTFE wear. Wear.

[15] Song F., Wang Q., Wang T. (2016). Effects of glass fiber and molybdenum disulfide on tribological behaviors and PV limit of chopped carbon fiber reinforced polytetrafluoroethylene composites. Tribol. Int..

[16] Amenta F., Bolelli G., Pedrazzi S., Allesina G., Santeramo F., Bertarini A., Sassatelli P., Lusvarghi L. (2021). Sliding wear behaviour of fibre-reinforced PTFE composites against coated and uncoated steel. Wear.

[17] Kandanur S.S., Rafiee M.A., Yavari F., Schrameyer M., Yu Z.-Z., Blanchet T.A., Koratkar N. (2012). Suppression of wear in graphene polymer composites. Carbon.

[18] Burris D.L., Sawyer W.G. (2006). Improved wear resistance in alumina–PTFE nanocomposites with regular shaped nanoparticles. Wear.

[19] Sun W., Liu X., Liu K., Xu J., Lu Y., Liu K. (2021). Mechanochemical functionality of graphene additives in ultralow wear polytetrafluoroethylene composites. Carbon.

[20] Xiao W., Ji X. (2021). Effect of nano fillers on the properties of polytetrafluoroethylene composites: Experimental and theoretical simulations. J. Appl. Polym. Sci..

[21] Bahadur S. (2000). The development of transfer layers and their role in polymer tribology. Wear.

[22] Harris K.L., Pitenis A.A., Sawyer W.G., Krick B.A., Blackman G.S., Kasprzak D.J., Junk C.P. (2015). PTFE tribology and the role of mechanochemistry in the development of protective surface films. Macromolecules.

[23] Nunez E.E., Polycarpou A.A. (2015). The effect of surface roughness on the transfer of polymer films under unlubricated testing conditions. Wear.

[24] Hu L., Zhong W., Dan J., Li Y., Yu Q. (2015). A study of the tribological behavior of transfer films of PTFE composites formed under different loads, speeds and morphologies of the counterface. Wear.

[25] Onodera T., Nunoshige J., Kawasaki K., Adachi K., Kurihara K., Kubo M. (2017). Structure and function of transfer film formed from PTFE/PEEK polymer blend. J. Phys. Chem. C.

[26] Gosvami N.N., Bares J.A., Mangolini F., Konicek A.R., Yablon D.G., Carpick R.W. (2015). Mechanisms of antiwear tribofilm growth revealed in situ by single-asperity sliding contacts. Science.

[27] Harris K.L., Curry J.F., Pitenis A.A., Rowe K.G., Sidebottom M.A., Sawyer W.G., Krick B.A. (2015). Wear Debris Mobility, Aligned Surface Roughness, and the Low Wear Behavior of Filled Polytetrafluoroethylene. Tribol. Lett..

[28] Ye J., Khare H., Burris D.L. (2014). Quantitative characterization of solid lubricant transfer film quality. Wear.

[29] Ye J., Khare H., Burris D.L. (2013). Transfer film evolution and its role in promoting ultra-low wear of a PTFE nanocomposite. Wear.

[30] Haidar D.R., Ye J., Moore A., Burris D.L. (2017). Assessing quantitative metrics of transfer film quality as indicators of polymer wear performance. Wear.

[31] Lin Z., Zhang K., Ye J., Gao B., Tao P., Zhang Z. (2023). Clarifying the importance of the running film to the ultra-low wear of the polymer composite by eliminating its individual effect. Tribol. Int..

[32] Urueña J.M., Pitenis A.A., Harris K.L., Sawyer W.G. (2015). Evolution and wear of fluoropolymer transfer films. Tribol. Lett..

[33] Alam K.I., Dorazio A., Burris D.L. (2020). Polymers tribology exposed: Eliminating transfer film effects to clarify ultralow wear of PTFE. Tribol. Lett..

[34] Sawyer W., Freudenberg K.D., Bhimaraj P., Schadler L.S. (2003). A study on the friction and wear behavior of PTFE filled with alumina nanoparticles. Wear.

[35] Wang Y., Yan F. (2006). Tribological properties of transfer films of PTFE-based composites. Wear.

[36] Zhang L., Xie T., Chen K., Li W. (2022). Observation and analysis of the terrace-like structured transfer film of SiO_2_/PTFE composites. Tribol. Int..

[37] Zhang L., Xie T., Chen K., Li C., Zhang J. (2021). Quantitative characterization of the transfer film morphology of SiO_2_/PTFE composite. Wear.

[38] Wahl K., Chromik R., Lee G.Y. (2008). Quantitative in situ measurement of transfer film thickness by a Newton’s rings method. Wear.

[39] Gu Y., Wang Z., Peng S., Ma T., Luo J. (2021). Quantitative measurement of transfer film thickness of PTFE based composites by infrared spectroscopy. Tribol. Int..

[40] Qi Y., Sun B.G., Wang H.G., Zhang Y., Gui G., Zhang P., Zheng X.B. (2023). Quantitative measurement of morphological characteristics of PTFE composite transfer films based on computer graphics. Materials.

[41] Otsu N. (1979). A threshold selection method from gray-level histograms. IEEE Trans. Syst. Man Cybern..

[42] Heath M., Sarkar S., Sanocki T., Bowyer K. (1998). Comparison of edge detectors: A methodology and initial study. Comput. Vis. Image Underst..

